# Longitudinal Imaging of the Skull Base Synchondroses Demonstrate Prevention of a Premature Ossification After Recifercept Treatment in Mouse Model of Achondroplasia

**DOI:** 10.1002/jbm4.10568

**Published:** 2021-11-09

**Authors:** Guylene Rignol, Stephanie Garcia, Florence Authier, Kaamula Smith, Lionel Tosello, Raphael Marsault, Pierre Dellugat, Diogo Goncalves, Marlene Brouillard, Jeffrey Stavenhagen, Luca Santarelli, Christian Czech, Elvire Gouze

**Affiliations:** ^1^ Rare Disease Unit Pfizer, Research and Development Nice France; ^2^ Bionea Biot France; ^3^ University of Dundee Dundee UK; ^4^ Université Côte d'Azur, CNRS, Inserm, iBV Nice France; ^5^ Therini Bio South San Franscico CA USA; ^6^ VectivBio Basel Switzerland; ^7^ InnoSkel Biot France

**Keywords:** ACHONDROPLASIA, CHONDROCYTE, FIBROBLAST GROWTH FACTOR RECEPTOR 3, RECIFERCEPT, SYNCHONDROSES

## Abstract

Achondroplasia is the most common form of short‐limb dwarfism. In this disorder, endochondral ossification is impaired due to gain‐of‐function mutation in the Fibroblast Growth Factor Receptor 3 (FGFR3) gene. In addition to short limbs, cranial base bones are also affected leading to shortening of the skull base and to serious neurological complications associated with foramen magnum stenosis. These complications are thought to be due to the delay or premature arrest of skull base growth, caused by an accelerated ossification of the sphenooccipital (SOS) and the intraoccipital (IOS) synchondroses. Skull synchondroses consist of two opposite growth plates sharing a common reserve zone of chondrocytes. In this study, we first characterized the skull base synchondroses ossification in a mouse model of achondroplasia carrying the human G380R mutation (*Fgfr3* ^*ach/+*^). We then addressed whether Recifercept, a soluble FGFR3, could prevent premature closure of these synchondroses. Postnatal radiological observations revealed the presence of bony bridge structures in one or more synchondroses in *Fgfr3* ^*ach/+*^ mice as early as postnatal day 3 in the most severe cases. The presence of early ossification correlated with the severity of the disease as it was associated with an arrest of the cranial base bone growth. Histological analyses indicated changes in the synchondroses structure and matrix proteoglycan contents confirming a process of ossification. Treatment of *Fgfr3* ^*ach/+*^ mice with Recifercept compared with vehicle prevented premature synchondrosis ossification and the transition to bone, resulting in improved skull shape and cranium ratio. Given the impact of Recifercept on synchondrosis inactivation, it is possible that it could prevent one of the most severe complication of achondroplasia if used early enough during bone development. These data support the clinical development of Recifercept for achondroplasia, and suggests that early treatment may be required to best address impaired endochondral bone growth. © 2021 The Authors. *JBMR Plus* published by Wiley Periodicals LLC on behalf of American Society for Bone and Mineral Research.

## 1 Introduction

Achondroplasia is the most common form of short‐limb dwarfism with a prevalence of 1 in 25,000 live births.^(^ ^1^ ^)^ In this disorder, endochondral ossification is impaired due to gain‐of‐function mutation of Fibroblast Growth Factor 3 (FGFR3), that leads to a decrease in chondrocyte proliferation and differentiation.^(^ ^2^ ^)^ In addition to short limbs, patients show craniofacial features including shorter antero‐posterior cranial base, prominent forehead and midfacial hypoplasia, spinal and foramen magnum stenosis.^(^ ^1^, ^3^ ^)^ These later can lead to serious neurological complications, brain stem compression, central sleep apnea, and in the most severe cases to death.^(^ ^4^, ^5^, ^6^ ^)^ Currently, surgeries such as enlargement of the foramen magnum or facial bones distraction are the only options for the clinical management of these complications.^(^ ^7^ ^)^

Craniofacial skeleton formation occurs through intramembranous and endochondral ossification processes.^(^ ^8^, ^9^ ^)^ In intramembranous process, mesenchymal cells directly differentiate in osteoblasts to form bone. In endochondral ossification, mesenchymal cells first differentiate into chondrocytes to form a cartilage template called the growth plate, which enlarges via chondrocyte proliferation. Thereafter, chondrocytes stop dividing and increase in volume to become hypertrophic. The surrounding matrix is then mineralized by osteoblasts leading to bone formation.^(^ ^8^, ^10^ ^)^ This process continues until full mineralization of the bone is completed and determines the height of the growth plate and allows linear bone growth.^(^ ^11^ ^)^ The cranial vault bones and the majority of the facial bones are formed intramembranously whereas bones from the skull base and the nasal septum are formed through endochondral ossification.^(^ ^10^, ^12^ ^)^ The cranial base is composed of parts of the occipital bone that surround the foramen magnum, the sphenoid, the petrous parts of the temporal bones, the ethmoid and the vomer.^(^ ^13^ ^)^ Bones of the skull base are separated by cartilage structure called synchondroses that include the sphenoethmoidal synchondrosis, intersphenoid synchondrosis (ISS), spheno‐occipital synchondrosis (SOS) and two intraoccipital synchondroses (IOS).^(^ ^14^ ^)^ Synchondroses consist of two opposite growth plates sharing a common reserve zone of chondrocytes and contribute to the elongation of the cranial base through endochondral process. Premature fusion of synchondroses can lead to abnormal development of the cranial base, the upper face and the cranial vault.^(^ ^15^, ^16^ ^)^

The skull base synchondroses and the SOS in particular have been described as having prominent role in growth of the human skull after birth^(^ ^17^ ^)^ and their premature closure is believed to be one of the factors that causes foramen magnum stenosis in achondroplasia due to shortening of the skull base.^(^ ^18^ ^)^ In average size children, the complete closure of the SOS shows evidence of the begining of fusion at a mean age of 7.5 years with complete fusion estimated to be between 15 and 20 years of age.^(^ ^19^ ^)^ The exact timing of premature closure of skull synchondroses in achondroplasia children is not known, but complete closure of some synchondroses has been observed as early as 1 year old.^(^ ^18^, ^20^ ^)^

In the first instance we performed a natural history of cranial base using a mouse model of achondroplasia carrying the human G380R mutation (*Fgfr3* ^*ach/+*^). We have characterized the timing and mechanism of inactivation of several sychondroses via ossification and their influence on skull shape, without any treatment. Recently, we have also developed a postnatal soluble FGFR3 (sFGFR3) therapy that counteracted the negative effect of FGFR3 signaling by restoring bone growth in an animal model of achondroplasia.^(^ ^21^ ^)^ Recifercept is an optimized version of sFGFR3 that is currently evaluated in phase 2 clinical trial. This study is the second publication with Recifercept treatment of mice.^(^ ^22^ ^)^ In the second part of this study, we evaluated the potential therapeutic effects of Recifercept in preventing the premature irreversible ossification of cranial base synchondroses using a longitudinal study design in which the same mice were analyzed at postnatal day 3, day 9 and day 22.

## 2 Materials and Methods

### 2.1 Animals

The study was divided into two parts, first, the natural history was performed on a set of animals and the treatment evaluation with Recifercept using a second set. All the experiments were performed on transgenic *Fgfr3* ^*ach/+*^ and WT FVB mice. This transgenic animal model essentially replicates the achondroplasia phenotype observed in patients.^(^ ^23^ ^)^ All animals were kept in a temperature and humidity‐controlled room, exposed to a 12‐hour light/dark cycle and had free access to standard laboratory food and water. Pups were kept with their genitors at all times. The Principles of Laboratory Animal Care (National Institutes of Health publication no. 85‐23, revised 1985; http://grants1.nih.gov/grants/olaw/references/phspol.htm) and the European commission guidelines for the protection of animals used for scientific purposes (http://ec.europa.eu/environment/chemicals/lab_animals/legislation_en.htm) were followed at all times. The study was approved by the local Institutional Ethic Committee for the use of Laboratory Animals (CIEPAL Azur) (approvals #05307.03 and APAFIS#9635‐2017032911018034 v4). Young *Fgfr3* ^*ach/+*^ mice can develop potential complications of achondroplasia.^(^ ^21^ ^)^ As such, during the first three weeks of life, individual mice were observed daily for the onset of bilateral paralysis or breathing problems that can lead to premature death. If difficulty in breathing or paralysis, indicated by bladder dysfunction, were observed, the animal was immediately euthanized. After weaning, the development of complications is very rare, and the phenotype is not harmful anymore. Animals were still observed every two days and any modification of behavior (such as prostration or absence of grooming) led to the immediate euthanasia of the animal. When appropriate, animals were sacrificed by lethal intraperitoneal injection of pentobarbital.

### 2.2 Natural history study of cranial base

*Fgfr3* ^*ach/+*^ mice were bred with WT mice to generate WT and heterozygous FVB animals. Animals were euthanized either at day 3 (n = 10 WT, n = 7 *Fgfr3* ^*ach/+*^ mice), or day 9 (n = 9 WT, n = 12 *Fgfr3* ^*ach/+*^ mice), or day 22 (n = 20 WT, n = 12 *Fgfr3* ^*ach/+*^ mice), to perform skull imaging, measurements and histology analysis.

### 2.3 Recifercept treatment

*Fgfr3* ^*ach/+*^ mice were bred with WT mice to generate WT and heterozygous animals. Whole litters were randomly allocated to treatment groups. As previously described,^(^ ^21^ ^)^ pups received from postnatal day 3 subcutaneous injections of either vehicle (phosphate buffered saline solution, n = 50 WT, n = 51 *Fgfr3* ^*ach/+*^ mice) or a 10 mg/kg Recifercept dose (n = 46 WT, n = 38 *Fgfr3* ^*ach/+*^ mice) twice a week for three weeks. Recifercept is an optimized version of the soluble FGFR3 and was prepared by culturing Clone #20‐6 from a GCHO cell line producing Recifercept in a single use 50 L bioreactor. Culture media containing Recifercept was purified by filtration and multiple chromatography steps. At postnatal day 22, after a total of six injections of Recifercept, mice were euthanized by intraperitoneal lethal injections of pentobarbital. Mice were genotyped at completion of the experiments by polymerase chain reaction as previously described.^(^ ^21^ ^)^

### 2.4 Skull imaging and measurements

All measurements were performed blinded to genotype and treatment by attribution of a unique 15‐digit number automatically to the radiograph which was different from the mice identification number. X‐ray images of the skulls in ventral position were acquired on a Faxitron® X‐ray radiographer (Edimex) using the following parameters: 26 kV and 19 s. The first (postnatal day 3) and second (postnatal day 9) skull X‐rays were performed under cold anesthesia, and the last one was performed after sacrification of the mice on postnatal day 22. The skull length, width, cranial base length, foramen magnum area, sternum length, floating rib and long bones (femur) were measured on X rays using open source software *Horos (horosproject.org)*. Body and tail lengths were measured using an electronic digital caliper.

### 2.5 Histology

Cranial base bones were fixed in 4% formalin solution for 24 hours, decalcified in 10% EDTA solution for 24 hours and paraffin‐embedded. Five‐micron sections were cut horizontally and stained with Alcian Blue (Sigma) for 30 minutes and counterstained with Nuclear Fast Red (Millipore).

For immunofluorescence analysis, sections were blocked 1 hour with 1.5% calf serum, incubated over night at 4°C with anti‐collagen X (Abcam, ab58632), anti‐heparan sulfate proteoglycan (Abcam, ab2501) or anti‐bone alkaline phosphatase (R&D, AF2909) primary antibodies and 1 hour at room temperature with appropriate Alexa Fluor secondary antibodies (1/1000 dilution) (Life Technologies, A11037). Nuclear staining was performed with Dapi (Millipore) and sections were imaged on an Axioplan2 upright microscope (Carl Zeiss Microscopy GmbH) with Zeiss 4X, 10X or 20X Achroplan dry NA 0.1.

### 2.6 Statistical analysis

Sample size and power calculation for each parameter were determined using the online calculator powerandsamplesize.com using the assumption that data follow normality, the significance level α equals to 0.05 and that the F‐test shows equal variances. Statistical analysis was performed using GraphPad Prism 7.0 software. D'Agostino & Pearson normality test, Shapiro–Wilk normality test, and KS normality test were performed to verify normality. For skeletal measurements that fulfilled normality and equal variance requirements, a one‐way ANOVA with a Dunnett's multiple comparison test (95% confidence interval to compare all the different groups) was performed. For skeletal measurements data sets that did not fulfill normality and equal variance requirements, a Kruskall‐Wallis test was performed. Mean values for each group were compared using the two‐tailed Student's test for comparisons of two independent groups. Survival was analyzed using a Kaplan Meier chart.

## 3 Results

### 3.1 *Fgfr3* ^*ach/+*^ mice display smaller skull and cranial base bones due to premature ossification of skull synchondroses

**Fig 1:**
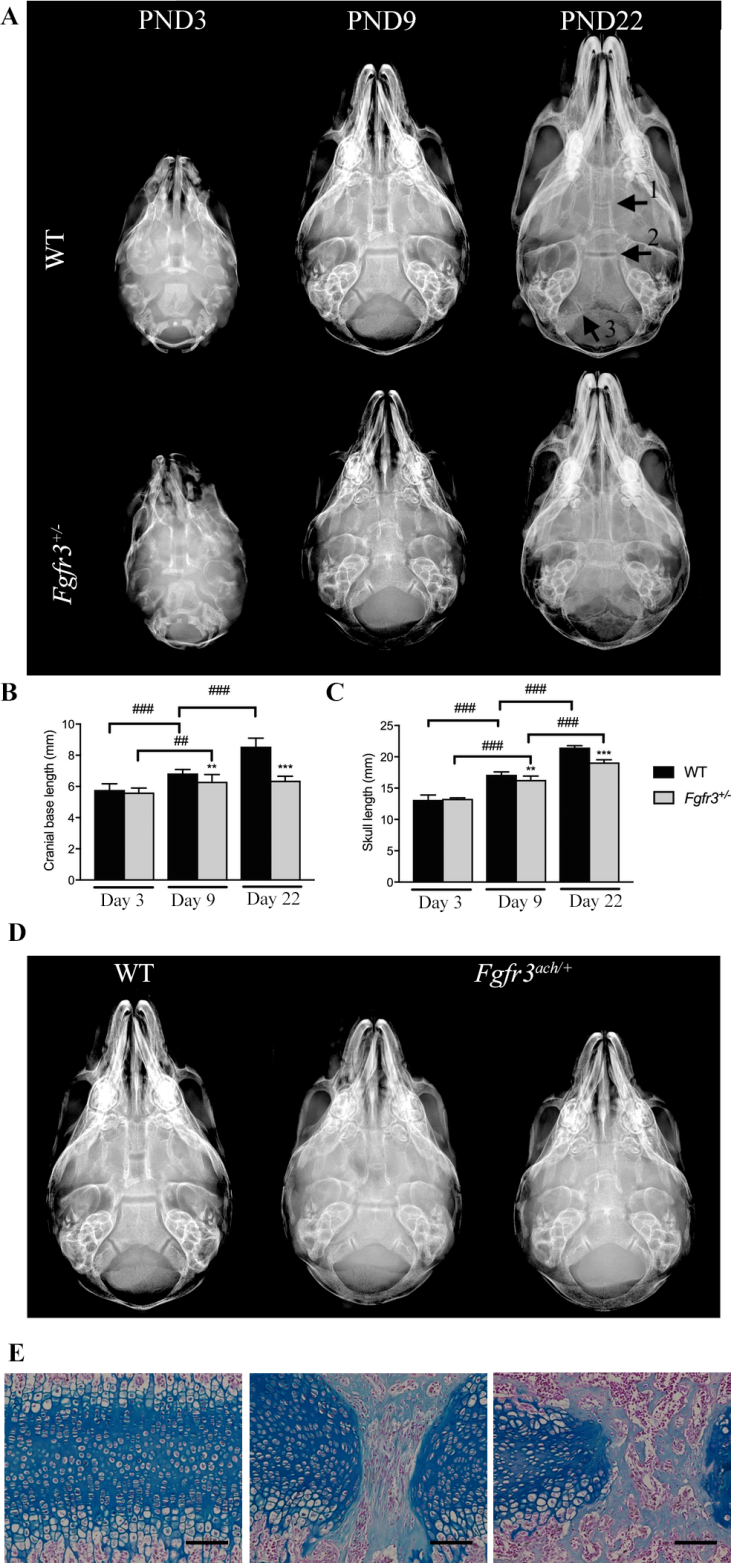
Premature closure of the SOS is associated with cranial base growth arrest in *Fgfr3* ^*ach/+*^ mice. (*A*) Representative radiological images of the skull of 3 (PND3), 9 (PND9), 22 (PND22) days old *Fgfr3* ^*ach/+*^ and WT mice. The different cranial base synchondroses are shown with numbered arrows (1: intersphenoid synchondroses [ISS]; 2: spheno‐occipital synchondrosis [SOS]; 3: intraoccipital synchondroses [IOS]). (*B*, *C*) Measurements of the cranial base length of 3, 9 or 22 days old WT and *Fgfr3* ^*ach/+*^ mice. (*D*) Whole skull ventral images of 9 days old WT and *Fgfr3* ^*ach/+*^ mice (*E*) Alcian blue staining of the SOS of 9 days old WT (left panel) and *Fgfr3* ^*+/−*^ mice (middle and right panels), ×10 magnification. ^##^ *p* < 0.005, ^###^ *p* < 0.001 compared to matching age and phenotype mice, ^*^ *p* < 0.05, ^***^ *p* < 0.001 compared to *Fgfr3* ^*ach/+*^ vehicle mice.

Achondroplasia patients suffer from short stature and significantly severe complications caused by skull deformities. To characterize the timing and mechanism of inactivation of several sychondroses a natural history study was performed on the skull of WT and *Fgfr3* ^*ach/+*^ mice at different age between postnatal day 3 and day 22 (Fig. 1). At postnatal day 3 (PND3), no difference in skull length or shape was observed between *Fgfr3* ^*ach/+*^ and WT animals, while at PND9 and PND22 *Fgfr3* ^*ach/+*^ mice exhibit shorter and rounder skull compared to their WT littermates (Fig. 1*A*). We subsequently investigated the timing of cranial base synchondroses ossification in *Fgfr3* ^*ach/+*^ mice. At PND3, *Fgfr3* ^*ach/+*^ pups did not show any sign of ossification of the synchondroses, while *Fgfr3* ^*ach/+*^ animals displayed exhibit partial ossification of the intersphenoid synchondrosis (ISS) and the two intraoccipital synchondroses (IOS). At PND9, we observed premature ossification at the center of the SOS with appearance of a bony bridge structure through the synchondrosis. At PND22, the ISS and IOS were completely ossified in all *Fgfr3* ^*ach/+*^ and a vaste majority of the SOS were ossified in *Fgfr3* ^*ach/+*^ mice. All cranial base synchondroses (ISS arrow 1, SOS arrow 2 and IOS arrow 3) remain cartilaginous with no sign of ossification at PND3, 9 and 22 in all WT animals (Fig. 1*A*).

To determine the relationship between premature synchondroses ossification and skull growth in WT and *Fgfr3* ^*ach/+*^ mice, cranial base lengths and skull lengths were measured either at PND3, PND9 or PND22. At PND3, no differences in either cranial base and skull lengths were observed between *Fgfr3* ^*ach/+*^ and WT mice (Fig. 1*B*,*C*). *Fgfr3* ^*ach/+*^ mice showed significant reduction in both cranial base and skull lengths at PND9 and PND22 compared to their WT littermates (Fig. 1*B*,*C*). While an increase of the cranial base length was observed between PND3, 9 and 22 in WT animals, the cranial base length was similar between PND9 and 22 in *Fgfr3* ^*ach/+*^ animals suggesting an arrest of the growth of the cranial base bone after PND9 (Fig. 1*B*). Skull lengths increased between PND3, 9 and 22 in both WT and *Fgfr3* ^*ach/+*^ animals, although the growth amplitude was reduced in *Fgfr3* ^*ach/+*^ animals (Fig. 1*C*). Altogether, these results show that premature ossification of the skull synchondroses is associated with an arrest of the cranial base bone growth and reduced skull growth leading to skull deformation in *Fgfr3* ^*ach/+*^ mice.

Interestingly, some variability in the SOS ossification status was observed within the *Fgfr3* ^*ach/+*^ mice population at PND9 (Fig. 1*D*) with animals showing an active unossified growth plate, an inbetween ossification stage with a bridge or an inactive, complete ossified synchondrosis. To further investigate the ossification status of the SOS, we performed histological analysis of the SOS from WT and *Fgfr3* ^*ach/+*^ mice at PND9. While WT animals exhibit normal organization of the SOS, *Fgfr3* ^*ach/+*^ mice display partial mineralization in the form of a bony like bridge structure in the middle of the SOS. This was associated with disorganization of the chondrocytes within the SOS and the loss of the columnar organization of the chondrocytes in the proliferative zone. This observation further confirms a premature ossification of the SOS in the *Fgfr3* ^*ach/+*^ animals (Fig. 1*E*). Interestingly, some *Fgfr3* ^*ach/+*^ pups exhibited denser and larger bony bridge structures within the SOS, suggesting an advanced state of ossification in those animals. This was associated with shorter and rounder aspect of the skulls in the corresponding animals (Fig. 1*E*) indicating that the degree of premature ossification could be associated with the severity of the craniofacial phenotype in the *Fgfr3* ^*ach/+*^ mice.

**Fig 2:**
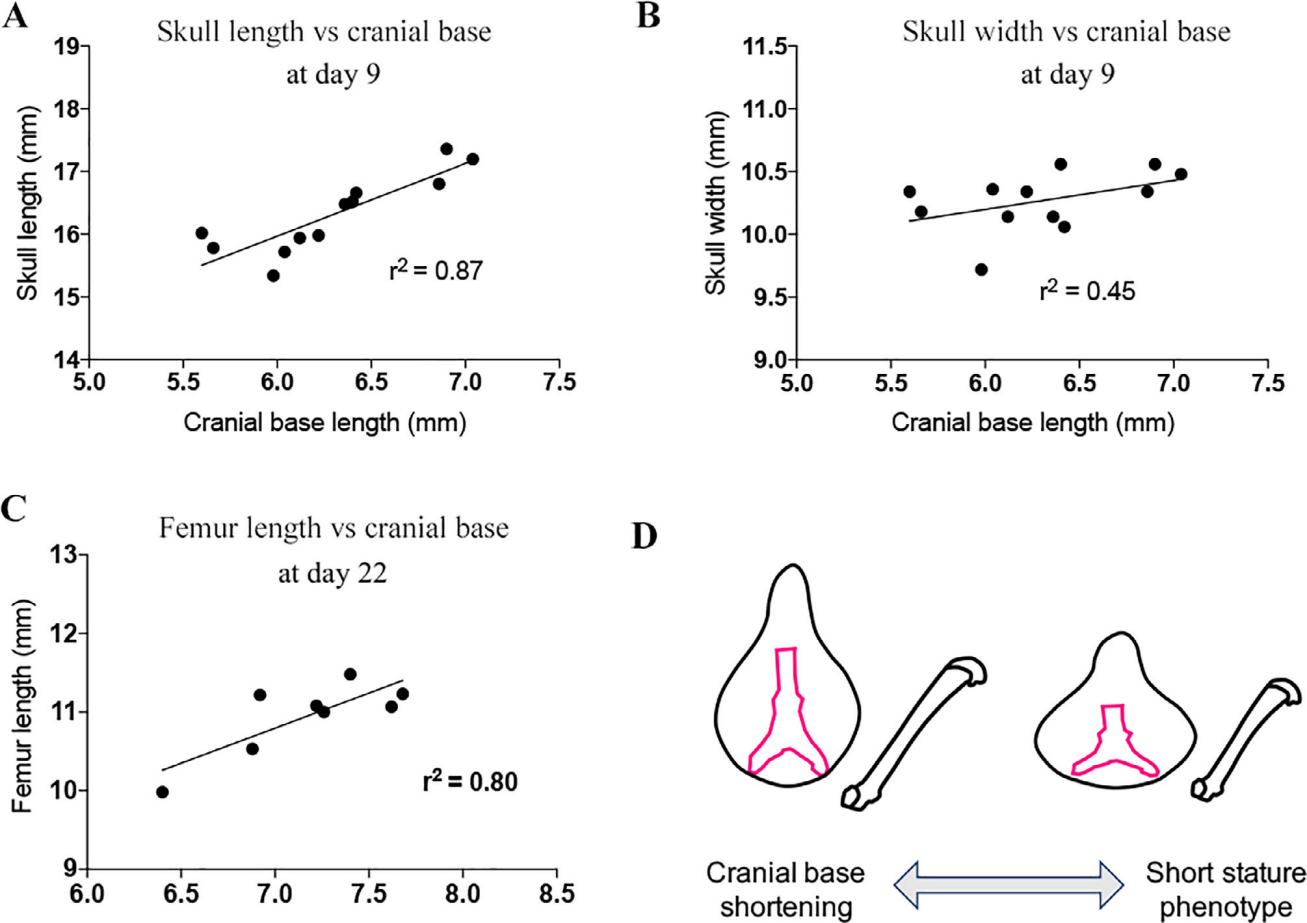
The degree of shortening of cranial base length is associated with skull length, width and femur length phenotype severity in *Fgfr3* ^*ach/+*^ mice. (*A*) Cranial base length versus skull length correlation in 9 days old *Fgfr3* ^*ach/+*^ mice. (*B*) Cranial base length versus skull width correlation in 9 days old *Fgfr3* ^*ach/+*^ mice. (*C*) Cranial base length versus femur length correlation in 22 days old *Fgfr3* ^*ach/+*^ mice. (*D*) Graphical representation of correlation between cranial base length and skull‐ and femur‐length phenotype.

To determine whether a more severe craniofacial phenotype could be an indicator of a severe short stature, we performed correlation analysis on skull measurements at PND9. We first observed that the cranial base length positively correlated with skull length (Fig. 2*A*) and did not correlated with skull width (Fig. 2*B*) in the *Fgfr3* ^*ach/+*^ mice. Interestingly, we also observed that the cranial base length strongly correlated with the femur length, used as an indicator of the short stature phenotype (Fig. 2*C*). These suggest that the shortening of the cranial base length is associated with the severity of the short stature phenotype in the *Fgfr3* ^*ach/+*^ mice (Fig. 2*D*).

Altogether, these suggest that *Fgfr3* ^*ach/+*^ mice display shortening of skull and cranial base bones lengths due to premature ossification of skull synchondroses.

### 3.2 Premature closure of SOS is associated with premature chondrocyte differentiation showing an earlier transition to an ossified structure in *Fgfr3* ^*ach/+*^ mice

**Fig 3:**
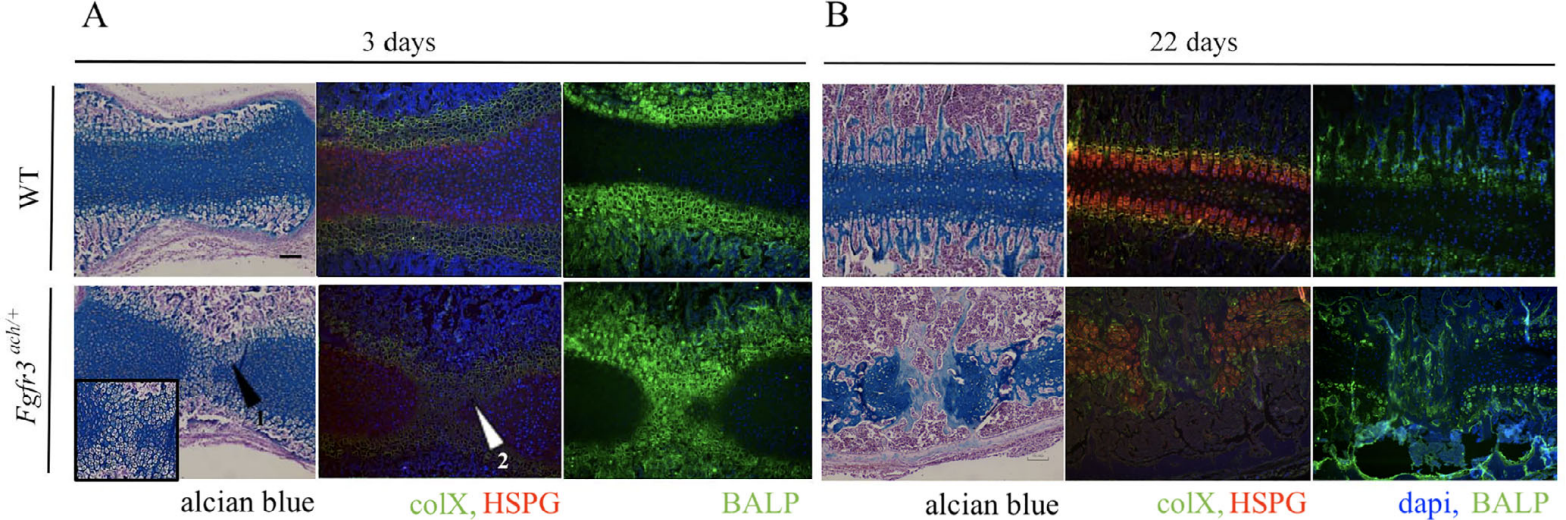
Premature closure of SOS is associated with alteration of chondrocytes arrangement. (*A*) Alcian blue staining, immunofluorescent collagen X (ColX) in green, heparan sulfate proteoglycans (HSPG) in red, dapi in blue and bone alkaline phosphatase (BALP) in green staining of the SOS of WT and *Fgfr3* ^*ach/+*^ at postnatal day 3 (PND3) and (*B*) postnatal day 22 (PND22). Scale bar is only represented on the upper left panel, the same ×10 magnification is applied to all pictures.

It is believed that in achondroplasia, constitutive FGFR3 signaling promotes premature synchondrosis inactivation/ossification in the cranial base by accelerating the transition from cartilage to bone of the skull sutures.^(^ ^24^ ^)^ To further investigate the mechanism initiating and underlying the premature ossification of the SOS in the *Fgfr3* ^*ach/+*^ mice, the matrix content of the cartilaginous stuctures were evaluated by histology at PND3 and PND22 in WT and *Fgfr3* ^*ach/+*^ mice (Fig. 3). At PND3 and PND22, WT animals exhibited normal chondrocyte organization with distinct reserve, columnar organized proliferation and hypertrophic zones within the SOS. Interestingly, changes in the SOS structure were observed as early as postnatal day 3 for some *Fgfr3* ^*ach/+*^ mice (Fig. 3). In these animals, as seen following alcian blue staining, narrowing of both chondrocyte resting and proliferative zones was observed due to the invasion of hypertrophic chondrocytes across the SOS (Fig. 3, arrow 1). Immunostaining for type X collagen (ColX) and heparan sulfate proteoglycans (HSPG) confirmed that these cells were indeed hypertrophic chondrocytes (arrow 2) and were secreting bone alkaline phosphatase (BALP) confirming an active process of mineralization at the future location of the bony bridge. By opposition, no sign of hypertrophic invasion was observed in the SOS of WT animals at PND3.

At PND22, the bony bridge structure through the SOS is composed of bone matrix that replaced the cartilaginous structure in *Fgfr3* ^*ach/+*^ animals, as shown by a blunt of ColX expression and a high content of BALP staining. These changes were also associated with a complete columnar disorganization of the proliferative chondrocytes expressing heparan sulfate proteoglycans (HSPG). Altogether, our results show that premature closure of the SOS is associated with premature chondrocyte differentiation showing an earlier transition to an ossified structure in *Fgfr3* ^*ach/+*^ mice.

### 3.3 Recifercept treatment restores skull shape in *Fgfr3* ^*ach/+*^ mice by preventing the complete ossification of the spheno‐occipital synchondroses

To study the effect of Recifercept therapy on cranial growth and subsequent skull shape, another subset of WT and *Fgfr3* ^*ach/+*^ mice were treated with either vehicle or 10 mg/kg Recifercept from postnatal day 3 twice a week for three weeks. We first verified that Recifercept had a similar effect on overall skeletal growth than the previous version of soluble FGFR3.^(^ ^21^ ^)^ While untreated *Fgfr3* ^*ach/+*^ mice were significantly smaller than WT littermates, Recifercept treatment resulted in a significant increase in body weight, body and tail length and survival versus the vehicle control, the latter correlated with the correction of rib cage deformities (Figure S1).

**Fig 4:**
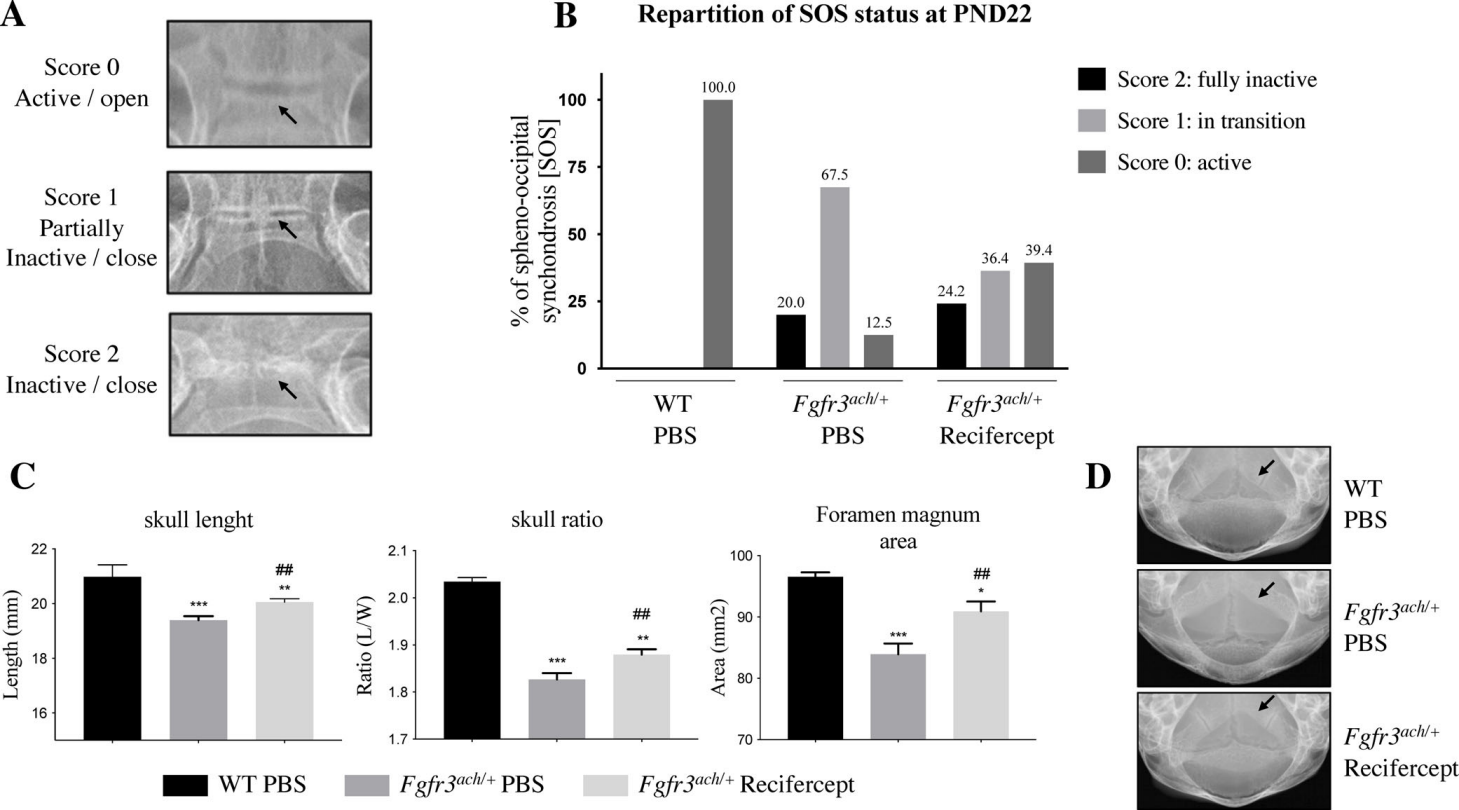
Recifercept restores skull shape in *Fgfr3* ^*ach/+*^ mice by preventing the premature closing/ossification of synchondroses. (*A*) Representative X‐ray imaging of synchondroses (SOS) status. Score 0 (active/ opened), score 1 (in transition/ partially closed) and score 2 (inactive = closed). Arrows indicate the location of the SOS (*B*) Repartition of synchondroses status after Recifercept and vehicle (PBS) treatment in WT and *Fgfr3* ^*ach/+*^ mice at postnatal day 22. (*C*) Skull measurements after Recifercept and vehicle (PBS) treatment in WT and *Fgfr3* ^*ach/+*^ mice at postnatal day 22. Recifercept significantly improves skull length, skull length/width ratio and foramen magnum area. (*D*) Representative X‐rays imaging of the foramen magnum area after Recifercept and vehicle (PBS) treatment in WT and *Fgfr3* ^*ach/+*^ mice at postnatal day 22. Arrows indicate the foramen magnum. ^#^ *p* < 0.05 compared with *Fgfr3* ^*ach/+*^ vehicle treated mice, ^*^ *p* < 0.05, ^***^ *p* < 0.001 compared to WT vehicle treated mice. (*B*).

To determine if Recifercept treatment was impacting the ossification process of the cranial base, we investigated the timing of cranial base synchondroses ossification in *Fgfr3* ^*ach/+*^ mice by performing longitudinal radiographic imaging of the skulls of WT and *Fgfr3* ^*ach/+*^ pups at PND3, PND9 and PND22 (Fig. 4, Table 1). A score was established to characterize the ossification status of the SOS as following: the SOS was active with no sign of ossification (no bridge: score 0), in transition with the appearance of an ossified bridge (partially inactive: score 1), or fully inactive (appeared completely ossified: score 2) (Fig. 4*A*). In both vehicle and Recifercept groups, all WT animals were scored 0 for all the timepoints (Table 1). At PND3, *Fgfr3* ^*ach/+*^ pups did not show score 2 for the SOS suggesting that the synchondrosis is active at that age. In *Fgfr3* ^*ach/+*^ animals treated with vehicle, the proportion of animals with a score 0 decreased over time indicating that the ossification process of SOS is progressing within the animal group. Very interestingly, the proportion of animals that displayed a score 0 remains stable over time in *Fgfr3* ^*ach/+*^ animals treated with Recifercept (Table 1). In addition, the proportion of score 0 synchondroses were higher with Recifercept treatment at PND 22 compared to vehicle treated control animals with 39.4% and 12.5% of animals respectively (Fig. 4 *B*, Table 1). These data strongly indicate that the premature ossification of the SOS is prevented by Recifercept treatment in these animals. At PND9, an increased number of Recifercept treated *Fgfr3* ^*ach/+*^ mice displayed a score 0 versus vehicle treated mice and overall proportions of score 0 and score 1 synchondroses were similar (Table 1). Altogether, Recifercept treatment is associated with a radiological improvement of spheno‐occipital synchondrosis score (Fig. 4*B*).

**Table 1:** Spheno‐occipital Synchondrosis [SOS] Status in Percentages of *Fgfr3* ^*ach/+*^ Mice Treated with Vehicle (PBS) and Recifercept Treatment at Postnatal day 3, day 9 and day 22. (score 0: active; score 1: partially inactive; and score 2: fully inactive)

%	Score	D3	D9	D22
WT PBS	0	100	100	100
	1	0	0	0
	2	0	0	0
*Fgfr3* ^*ach/+*^ PBS	0	55	30	12.5
	1	45	70	67.5
	2	0	0	20
WT	0	100	100	100
Recifercept	1	0	0	0
	2	0	0	0
*Fgfr3* ^*ach/+*^	0	42.4	39.4	39.4
Recifercept	1	57.6	60.6	36.4
	2	0	0	24.2

As a consequence, treatment with Recifercept resulted in a significant mitigation of the cranial phenotype observed in *Fgfr3* ^*ach/+*^ mice (Fig. 4*C*,*D*). Skull length, length/width skull ratio and foramen magnum area were all improved after a 3‐week treatment with Recifercept. The cranial phenotype of WT animals was not impacted by Recifercept, as 100% of synchondroses are active at postnatal day 22 in these animals.

Altogether these results suggest that before ossification is complete, it is possible to prevent completion of the ossification process of synchondroses that are transitioning to a bony structure. Thus, even though the skull deformities are already visible at birth, an early Recifercept treatment has the potential to prevent the premature closure of the cranial base synchondroses in children with achondroplasia.

## 4 Discussion

In addition to short stature, achondroplasia patients suffer from severe skull deformities and subsequent complications.^(^ ^1^ ^)^ On top of the characteristic facies that trigger important social stigmata, the most common complications are ENT (ear, nose, and throat) issues including frequent ear infections and obstructive sleep apnea. Because of the skull growth arrest, stenosis of the foramen magnum can be observed. This has been involved in increased sudden death syndrome in infants less than a year old, and also frequently lead to hydrocephaly.^(^ ^18^ ^)^ In designing a new effective treatment for achondroplasia, it is thus essential to restore skull development to prevent the occurrence of these complications. In this study we thus aimed to characterize the impact of Recifercept, a novel therapy based on the extracellular domain of human FGFR3,^(^ ^22^ ^)^ on the premature closure of the cranial base synchondroses in a mouse model of achondroplasia carrying the G380R mutation in the FGFR3 gene. A key and novel finding from this study, that as far as we are aware has not been reported previously, is that Recifercept can both prevent premature inactivation (ossification), and in some cases prevent the complete transition to bone, of cranial synchondroses. To demonstrate this, longitudinal follow up using non‐invasive X‐ray imaging technique was performed on the same animals at postnatal days 3, 9 and 22. This allowed the evaluation of Recifercept effect on the ossification of the cranial base synchondroses. The synchondroses are cartilage structures separating bone growth plates that are responsible for the lengthening of the cranial base through endochondral ossification. Once closed, the synchondrosis is irreversibly ossified thus inactive, stopping further cranial base lengthening and resulting in abnormal development of the upper face and the cranial vault. By delaying closure and reversing partial closure of synchondroses, Recifercept alleviates the adverse impact of achondroplasia on skull development. Using tyrosine kinase inhibitors acting directly on the FGFR3 signaling, a similar effect on foramen magnum has been reported,^(^ ^25^ ^)^ suggesting that, if well tolerated, a drug that acts directly on FGFR3 has the potential to impact the facies phenotype in achondroplasia. Our findings also extend previous analyses, which indicated that sFGFR3 could increase chondrocyte proliferation and differentiation and improve bone growth in mice,^(^ ^21^ ^)^ as well as prevent the atypical obesity associated with achondroplasia in mice.^(^ ^26^ ^)^

While these novel findings are in mice, and need to be confirmed in children diagnosed with achondroplasia, the prevention of the SOS premature ossification with Recifercept offers the possibility of being able to prevent or alleviate severe complications of achondroplasia if it is used early enough during bone development. In two studies, premature closure of the SOS was reported in heterozygous achondroplasia children of 1 and 2 years old.^(^ ^18^, ^20^ ^)^ Observation of one achondroplasia homozygous neonate revealed closure of all cranial base synchondroses. Partial inactivation of the SOS was noticed in a fetus of approximately 40‐weeks' gestation suffering from thanatophoric dysplasia type I (considered to be a severe achondroplasia phenotype).^(^ ^27^ ^)^ These data suggest a correlation between the severity of the skeletal phenotype and the early inactivation of the cranial base synchondroses. Additionally, they indicate that if Recifercept was to be used in the clinical setting, it would need to be initiated in early childhood before irreversible inactivation and ossification of cranial base synchondroses occurs in order to ameliorate the effects of achondroplasia on skull shape.

In the natural history part of our study, we observed the premature inactivation of the SOS starting at postnatal day 9 in *Fgfr3* ^*ach/+*^ pups and increasing by day 22 with the appearance of a bridge structure across from the SOS. Premature inactivation of synchondroses in the cranial base has also been shown in other mice models of G369C, S365C and G374R FGFR3 mutations.^(^ ^27^, ^28^, ^29^ ^)^ In *Fgfr3* ^*ach/+*^ mice, Recifercept treatment increased the proportion of active synchondroses at postnatal day 9 and day 22 versus vehicle control, although the overall proportion of active and transitioning synchondroses was similar between treatment groups at PND9. Importantly, the longitudinal radiographic analysis revealed that compared to the vehicle, Recifercept was able to reduce the overall proportion of transitioning synchondroses between postnatal day 3 and day 22. This improvement in synchondroses inactivation was associated with improvements in the skull phenotype measured by increases in skull width to length ratio and foramen magnum area.

In conclusion, Recifercept treatment was associated with a wide range of improvements in *Fgfr3* ^*ach/+*^ mice including significantly reduced mortality, increased body weight and improved skeletal bone development. The premature inactivation of the synchondroses is associated with impaired growth of the cranial base bones in *Fgfr3* ^*ach/+*^ mice. Recifercept improved bone growth of the cranial base in *Fgfr3* ^*ach/+*^ mice before the full inactivation of the SOS. This was associated with improvements in skull length and the foramen magnum area and restored the cranium ratio and shape of the skull. Thus, this therapeutic strategy might be effective to restore impaired craniofacial development including midface hypoplasia observed in achondroplasia patients.

## Conflict of Interest

Guylène Rignol, Diogo Gonçalves, Pierre Dellugat, Raphael Marsault and Christian Czech are employees of Pfizer. Kaamula Smith, Florence Authier, Lionel Tosello, Marlene Brouillard, Stephanie Garcia, Jeffrey Stavenhagen, are former employees of Therachon. Elvire Gouze is a former consultant of Therachon. Therachon is a wholly owned subsidiary of Pfizer.

## Editorial support

The support provided by Marcella DeSimone at Caudex, funded by Pfizer, consisted solely of manuscript formatting, and no contribution was made to editorial content.

5

### Peer Review

The peer review history for this article is available at https://publons.com/publon/10.1002/jbm4.10568.

## Supporting information


**Figure S1.** **In *Fgfr3*** ^***ach/+***^ **mice, Recifercept significantly improves skeletal development inkling** (*A*) body weight, (*B*) body length and (*C*) tail length versus vehicle at postnatal day 22. Recifercept also improves survival (*D*) and rib cage complications as seen by costal cartilage length (*E*) and sternum development (*F*) versus vehicle in WT and *Fgfr3* ^*ach/+*^ *mice*. Untreated *Fgfr3* ^*ach/+*^ mice showed an abnormal sternum development as seen by the absence of lower ossification centers (arrow). Data are the mean ± standard error mean (SEM) and followed normal distribution. # *p* < 0.05 compared to *Fgfr3* ^*ach/+*^ vehicle mice, *** *p* < 0.001 compared to WT vehicle mice.
