# Supplementary figures and images for: Longitudinal Imaging of the Skull Base Synchondroses Demonstrate Prevention of a Premature Ossification After Recifercept Treatment in Mouse Model of Achondroplasia
Source: JBMR Plus. 2021 Nov 9;6(2):e10568. doi: 10.1002/jbm4.10568 (PMC8861980; doi:10.1002/jbm4.10568)

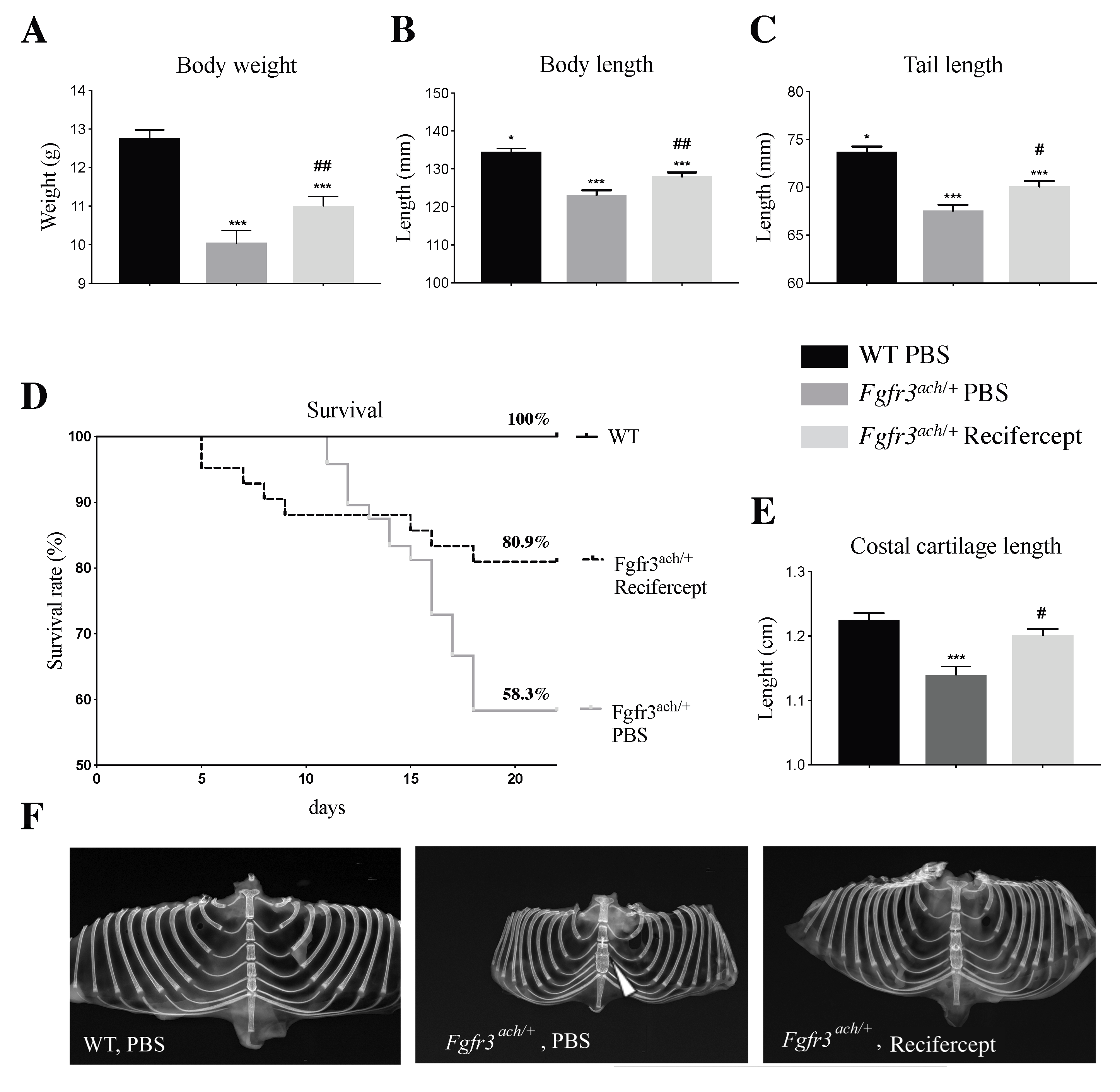

Supplement: Supplementary file 1 — Figure S1. In Fgfr3 ach/+ mice, Recifercept significantly improves skeletal development inkling (A) body weight, (B) body length and (C) tail length versus vehicle at postnatal day 22. Recifercept also improves survival (D) and rib cage complications as seen by costal cartilage length (E) and sternum development (F) versus vehicle in WT and Fgfr3 ach/+ mice. Untreated Fgfr3 ach/+ mice showed an abnormal sternum development as seen by the absence of lower ossification centers (arrow). Data are the mean ± standard error mean (SEM) and followed normal distribution. # p < 0.05 compared to Fgfr3 ach/+ vehicle mice, *** p < 0.001 compared to WT vehicle mice. [file JBM4-6-e10568-s001.tiff]
